# Durable Resistance to Crop Pathogens: An Epidemiological Framework to Predict Risk under Uncertainty

**DOI:** 10.1371/journal.pcbi.1002870

**Published:** 2013-01-17

**Authors:** Giovanni Lo Iacono, Frank van den Bosch, Chris A. Gilligan

**Affiliations:** 1 Department of Veterinary Medicine, Disease Dynamics Unit, University of Cambridge, Cambridge, United Kingdom; 2 Rothamsted Research, Harpenden, United Kingdom; 3 Epidemiology and Modelling Group, Department of Plant Sciences, University of Cambridge, Cambridge, United Kingdom; Plant Pathology Department, IFAS University of Florida, United States of America

## Abstract

Increasing the durability of crop resistance to plant pathogens is one of the key goals of virulence management. Despite the recognition of the importance of demographic and environmental stochasticity on the dynamics of an epidemic, their effects on the evolution of the pathogen and durability of resistance has not received attention. We formulated a stochastic epidemiological model, based on the Kramer-Moyal expansion of the Master Equation, to investigate how random fluctuations affect the dynamics of an epidemic and how these effects feed through to the evolution of the pathogen and durability of resistance. We focused on two hypotheses: firstly, a previous deterministic model has suggested that the effect of cropping ratio (the proportion of land area occupied by the resistant crop) on the durability of crop resistance is negligible. Increasing the cropping ratio increases the area of uninfected host, but the resistance is more rapidly broken; these two effects counteract each other. We tested the hypothesis that similar counteracting effects would occur when we take account of demographic stochasticity, but found that the durability does depend on the cropping ratio. Secondly, we tested whether a superimposed external source of stochasticity (for example due to environmental variation or to intermittent fungicide application) interacts with the intrinsic demographic fluctuations and how such interaction affects the durability of resistance. We show that in the pathosystem considered here, in general large stochastic fluctuations in epidemics enhance extinction of the pathogen. This is more likely to occur at large cropping ratios and for particular frequencies of the periodic external perturbation (stochastic resonance). The results suggest possible disease control practises by exploiting the natural sources of stochasticity.

## Author Summary

We want to understand if, and how, the evolution of a pathogen can be delayed/accelerated by random fluctuations always occurring in epidemics. We studied a simple biological system relevant to agriculture: a resistant crop immune to the disease, and a plant pathogen that defeats the resistance after a single mutation. Eventually the population of these more harmful pathogens will take over and the resistance can no longer protect the crop. As the availability of such resistant genes is limited in nature, this is an important problem to ensure food security for future generations as well as reduction in pesticide usage. We used a mathematical model to show that in general large stochastic fluctuations in epidemics enhance extinction of the pathogen, especially of the emerging mutant strains. We know that periodically forced epidemics oscillate at larger amplitude at some frequencies than at others (resonance), then by adequately perturbing the system (e.g. by alternating different types of fungicides) we can cause massive fluctuations in the small pathogen population increasing the chances of extinction. If such hypotheses will be experimentally confirmed, we could alleviate the disease, reduce chemical control, and in general, mitigate the risk of developing highly harmful pathogens (*e.g.* superbugs insensitive to antibiotics).

## Introduction

There is increasing social pressure to integrate science, policy and regulation in order to assess and minimize the risks associated with agricultural practices. Major risks and uncertainties persist whereby pests and pathogens rapidly overcome disease control methods using resistant cultivars and fungicides. Although disease resistant genes have been successfully used for disease management, many crop geneticists and plant breeders view resistance genes as a limited and potentially non-renewable resource, whereby once a pathogen has evolved to overcome the resistance, the resistance genes have permanently lost their value. Thus one of the key goals of virulence management is to increase the durability of crop resistance, a concept that has been extensively discussed in the literature, but which is still difficult to measure and predict [1]–[7]. Johnson [2] was perhaps the first to provide a definition of durable resistance, *i.e.* a resistance that remains effective over a prolonged period of widespread use under conditions conducive to the disease. However, such definition, although conceptually simple, does not provide an objective procedure for measuring and predicting the durability of crop resistance (see *e.g.* the discussion in [3]). In particular, the beguilingly simple concepts of ‘remaining effective’, ‘prolonged period’ and ‘widespread use’ are subject to a range of interpretations. Durability of resistance is also confounded with the inherent variability exemplified by a wide range of plant pathogens. As pointed out by Leach *et al.* [3], although many resistance genes have been identified in plant germplasm, identifying the factors that render the resistance ‘effective’ is still a challenging task. One exception is, perhaps, the polygenic *vs* monogenic paradigm, according to which resistance due to the additive action of many genes (also known approximately in the literature as polygenic, quantitative, horizontal resistance, see *e.g.* [8], [9]) is expected to be more durable than resistance due to the action of a single gene (also referred to as monogenic, qualitative, vertical resistance [1], [4]–[10]). However, even this generally accepted consensus has been challenged by several authors showing that erosion of polygenic resistance may be important and relatively rapid [2], [11]–[19] and presenting evidence of durable resistance due to the action of a single gene [2], [18].

This raises the key question why resistance, especially monogenic resistance, can be so ephemeral and subjected to the well known boom-and-bust cycles [20]. The review of Leach *et al.* [3] focuses on this issue and supports the hypothesis that the inherent quality and durability of a plant resistance gene is a direct function of the amount of fitness penalty imposed on the pathogen to overcome that resistance gene. Despite this important clarification, the mechanism regulating the durability of resistance is expected to be more complex than the simple molecular changes alone in pathogen adaptation and any associated fitness cost. The population sizes of the pathogen and host also matter. McDonald and Linde [20] provide a conceptual overview of the evolutionary forces that drive the evolution of plant pathogens (mutation, genetic drift, gene flow, reproduction/mating system, and selection) emphasising the role of population size. A large population is likely to have greater gene diversity than a smaller population and it can influence the so-called random genetic drift (*i.e.* the change in the frequency of alleles in a randomly chosen subset of a population) that typically occurs when a subset of population survives a catastrophic event that dramatically reduces the population size (a bottleneck), or due to external immigration of a small, random subset of a pathogen into a new host. In addition, the durability of resistance may be affected by the landscape composition (*i.e.* host variety frequencies) [21] and can be increased by using spatially heterogeneous mixtures of different cultivars with similar agronomic traits, but differing in resistance genes [5], [17], [22]. All these studies suggest that any theoretical approach to analyse the evolution of pathogen and durability cannot focus on gene frequency alone (the proportion of the pathogen population carrying a particular allele of a gene) and the density of the host and the pathogen ought to be included.

**Figure 1:**
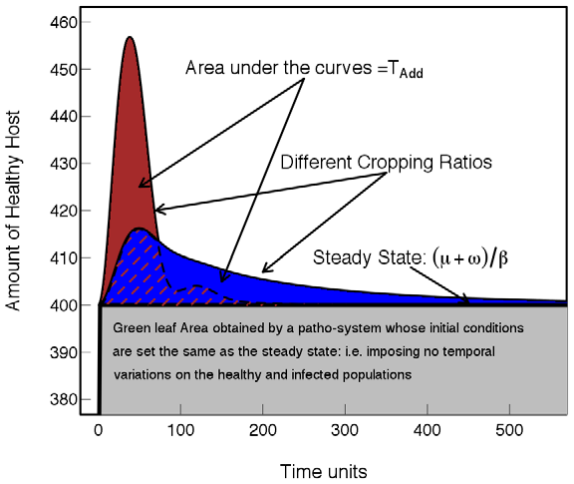
Illustration of measurement of durability of resistance. When the resistance is broken the resistant and susceptible cultivars are no longer distinguishable from each other; the pathosystem approaches the same equilibrium state as if the resistant cultivar was never deployed. The durability of resistance corresponds to the extra amount of healthy host gained, due to the resistant genes, during this time interval. This measure corresponds to the area 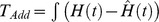 *dt*; *H(t)* is the healthy host and 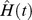 the steady state, *i.e.* when the system is no longer subjected to temporal variations. Two explicative cases with different cropping ratios are shown. In the deterministic model the two areas are expected to be the same.

To this end van den Bosch and Gilligan [23] explicitly linked population dynamics and population genetics to investigate the durability of resistance. They introduced new concepts to measure durable crop resistance and analysed these using deterministic models. They identified three measures, *the expected time until a virulent genotype invades* following release of a resistant crop cultivar, the *time until a virulent genotype takes over* the pathogen population and the *additional number of uninfected host growth days*, 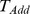, effected by the growth of the resistant cultivar (see Figure 1). For each measure they examined the effect of cropping ratio (the proportion of resistant cultivar grown in a landscape) on the durability of resistance. Here we focus on 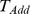 because of its practical usefulness in measuring durability of resistance and its applicability to pesticides, antibiotics and drug resistance (see also [24], [25]). To a first approximation 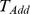 can be identified with the additional crop yield gained during the deployment of a resistant gene [26]. Thus in the light of Johnson's [2] definition, the resistance is considered to ‘remain effective’ until its use (deployment period) continues to produce additional crop yield; as the frequency of the virulent strain increases, the contribution to the additional crop decreases to zero. In this case the resistance is considered broken and the resistant and susceptible cultivars are no longer distinguishable from each other. Thus any further deployment of the resistance gene in a resistant cultivar has no effect.

A key message from the work of van den Bosch and Gilligan [23], also consistent with the predictions of Bonhoeffer [24] for antibiotic management, is that the durability of resistance is unaffected by the cropping ratio. van den Bosch and Gilligan [23] proposed the following explanation: increasing the proportion of resistant crop initially decreases the total pathogen population, but increases the selection pressure on the pathogen and consequently the resistance is more rapidly broken down [20]. The two effects tend to compensate and the total gain is unaltered, *i.e.* giving the same areas for deployment of resistant cultivars under different cropping ratios as shown in Figure 1. It is unlikely, however that in the field the solution is truly as simple as analysis of the deterministic model suggests, not least because the model [23] ignores important sources of variability, such as environmental and demographic stochasticity, rendering prediction of limited value [27].

Despite a growing body of research focused on stochastic disease dynamics, the role of demographic and environmental stochasticity on the dynamics of an epidemic is still not fully understood. For example, can we quantify the effect of random noise on the evolutionary forces classified by McDonald and Linde [20]? Does stochasticity delay/accelerate the evolution of pathogens? If so, how? To our knowledge, there is no theoretical framework available that investigates the effects of demographic and environmental stochasticity on the *evolution of the pathogen* and thus the *durability of crop resistance*. Here, building on previous work of van den Bosch and Gilligan [23] we now take account of variability. More precisely we formulate a stochastic, mathematical model to test the following hypotheses, that:

- in contrast with the finding of van den Bosch and Gilligan [23], the durability of crop resistance depends on the cropping ratio when we take account of demographic stochasticity.
- a superimposed external source of stochasticity (*e.g.* due to environmental factors) interacts with the intrinsic demographic fluctuations and affects the durability of resistance.

We show that, for the pathosystems considered here, large stochastic fluctuations, particularly at the beginning of epidemics, enhance extinction of the pathogen, and especially the virulent strain, so promoting the durability of resistant cultivars. This has important theoretical consequences as it shows that the evolution of pathogens is directly affected by demographic and environmental stochasticity. The findings also suggest possible disease control practises by exploiting natural sources of stochasticity.

## Materials and Methods

**Figure 2:**
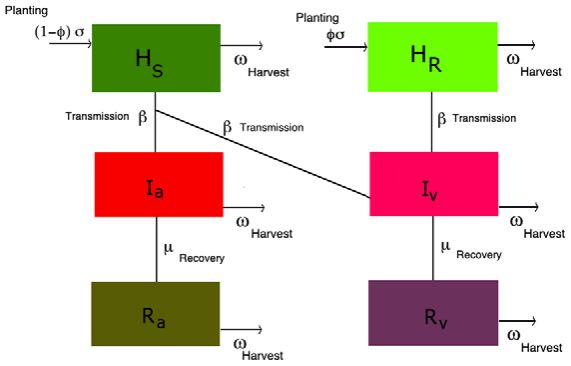
Stochasticity and life-cycle parameters. SIR model for two cultivars and two strains (avirulent and virulent) of the pathogen. The model does not explicitly compute the population of the pathogen but the infected categories *I_a_* and *I_v_*, representing the host populations (irrespective of being susceptible or resistant), infected by the avirulent and virulent strains, which generate new pathogens. For visual purposes the small effect of mutations is not illustrated in the diagram.

Our models are motivated for a broad range of crops and plant pathogens. The target hosts and pathogens are typified by cereal rusts and mildews but are by no means restricted to these. We used a SIR epidemic model to study a system comprising two cultivars (susceptible and qualitatively resistant) and two pathogen strains (virulent and avirulent). The unit of interest may be a plant but more usually it will be a unit of susceptible tissue such as a leaf or part thereof. In this non-spatial model, the populations of individuals are homogeneously mixed. The pathogen is transmitted from individual to individual when ‘encounters’ occur (*e.g.* a spore depositing on healthy tissue) with transition probability proportional to the number of possible encounters. The transmission of infection is described by a process like: 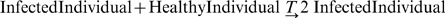, where *T* is the transmission probability from one category to another. This system can be seen as a birth-death system with many variables [28]. By writing down all possible transitions from one category to another with the adequate transition probabilities we obtain a Master Equation (ME) [28], [29]. High levels of accuracy and realism are possible by using models based on the solution of the Master Equation. However, for large populations the mathematics become intractable. A compromise is represented by approximating the ME by invoking the Kramer-Moyal expansion [28]. The approximation leads to a Fokker-Planck equation (FPE) providing the coefficients for the Langevin equation which can be used to simulate individual stochastic realisations (see Supporting Information):
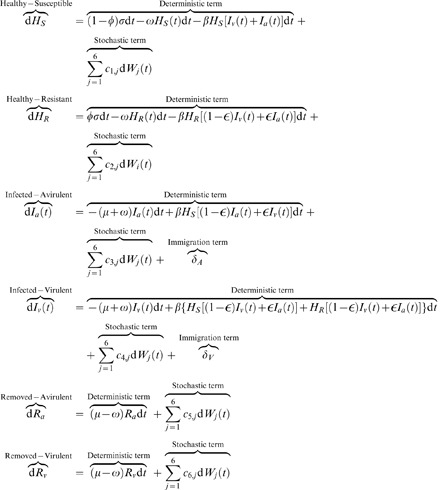
(1)where 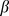 is the transmission rate; 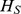 and 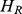 are the densities of susceptible (healthy) and resistant hosts; 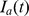 and 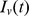 are the densities of infected hosts by the virulent and avirulent pathogen; 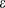 the rate of mutations; 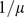 the infectious period; 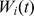 is a Wiener process with mean zero and variance 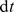 [28], [29]; the terms 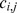 are the entries of the diffusion matrix in the corresponding FPE, they depend solely on the state of the system and on parameters used as model input. We also superimpose that the susceptible and resistant cultivar increase continuously with rates 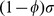 and 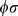 respectively, where 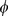 is the fraction of resistant crop and 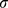 is a constant planting rate. Both hosts are harvested with rate 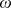. The pathosystem is illustrated in Figure 2. The model therefore applies to a system of continuous harvesting and sowing, typified by the management of continuous cropping systems, common in tropical regions. We have also chosen this system to enable comparison with other epidemiological models, where individuals are born with a constant birth rate 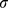 and a proportion of the entire population dies with rate 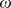, analogous to the systems of Bonhoeffer and Mclean [24], [25] for antibiotics and vaccination management. The continuous (non-seasonal) formulation of the pathosystem allows the analysis of periodic disturbance on the typical frequencies of the system (see below) that are unconfounded by seasonality. It also simplifies the mathematical analysis.

In the current paper planting and harvesting are assumed to be fully farmer-controlled and therefore not subjected to stochastic fluctuations. Immigration of avirulent and virulent pathogens from an external source are also included and modelled as a Poisson process with mean 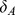 and 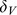 for the avirulent and virulent strains, respectively. Unless otherwise stated the values of the parameters are shown in Table 1. As we can see the deterministic term is formally the same as the deterministic model of Mclean [25] and compatible with the model of van den Bosch and Gilligan [23] with mutations. From the system of Langevin equations we can calculate the additional number of uninfected host growth days:
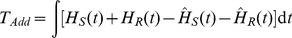
(2)where 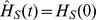 and 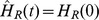 are the steady states for the susceptible and resistant host (*i.e.* the solutions of equation 1 when the derivatives on the lhs of equations and the stochastic terms are set to zero). When both pathogen strains are present, the system reaches an equilibrium in which the virulent genotype coexists with the resistant and susceptible cultivars and the avirulent genotype goes extinct [23]. As shown by [23] 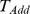 is interpreted as a measure of the durability of crop resistance. These methodologies result in many stochastic time-series for the population of infected and healthy hosts. For each single stochastic realisation, we calculated the durability 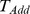 and how this depends on parameters, such as the cropping ratio. By using the wavelet analysis [30], a suitable tool for transient regimes like the current one, we have identified the dominant frequencies of the system. Then we replaced the fixed life-cycle parameters in the model with periodically variable parameters. Underlying this approach is the assumption that external factors such as seasonality have an immediate effect on the population. Here we tested hypotheses i) and ii) by:

**Table 1:** Parameters.

State Variable and Parameter	Symbol and Value (in adequate units)
Healthy, susceptible, host	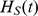
Healthy, resistant, host	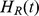
Host infected by the avirulent strain	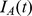
Host infected by the virulent strain	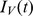
Removed host that was infected by the avirulent strain	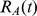
Removed host that was infected by the virulent strain	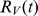
Initial basic reproductive ratio	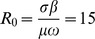 ( 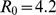 Figures 3,4,5)
cropping ratio 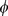	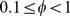
Planting ratio	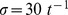 ( 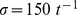 Figures 3,5)
Harvest rate	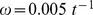
Transmission rate	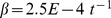 ( 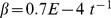 Figures 3,5)
Deployment time	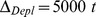 ( 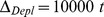 Figures 5)
Infectious Period	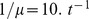 ( 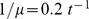 Figures 3,5)
Rate of mutations	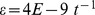
Immigration of avirulent pathogens	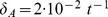 , ( 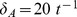 Figure 5)
Immigration of virulent pathogens	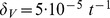 , ( 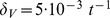 Figure 5)
Number of stochastic realizations	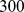
Initial density of susceptible healthy, avirulent and virulent host	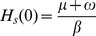 , 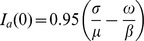 , 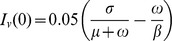 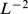

Values of the parameters used in the simulations. The dimensions of the parameters are expressed in terms of time units 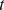 (*e.g.* measured in days) and length units 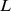.

- simulating many stochastic realisations according to equation (1) and calculating the durability of resistance according to equation (2) for a range of values of the cropping ratio. The results were compared with the deterministic counterpart when all the term 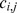 in the diffusion matrix are set to zero.
- repeating the procedure in i) as well as imposing a periodic perturbation on the system. This was done by a regular variation of the value of one of the life-cycle parameters. The periodic perturbations were always in phase for all random realisations. This scenario mimics, for instance, a regular variation of the environmental conditions or a continuous application of fungicides when the mean dosage is altered in a periodic fashion. The results were compared with the scenario when none of the parameters were varied.

## Results

**Figure 3:**
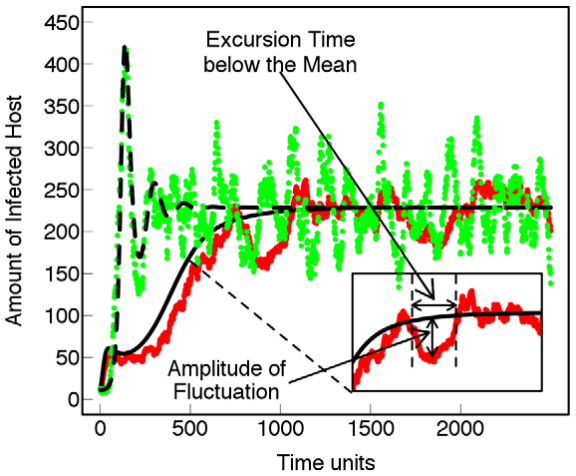
Stochasticity and life-cycle parameters. Deterministic (black lines) and stochastic profiles for the amount of infected host with dfferent life-cycle parameters. Black dashed line and green dots: results for a simulated process with parameters as in in Table 1. Black continuous line and red line: *σ* and *μ* increased by a factor five; in both cases no mutations and no external immigration of pathogen. *β* = 0.7*E*−4 *t* ^−1^.

**Figure 4:**
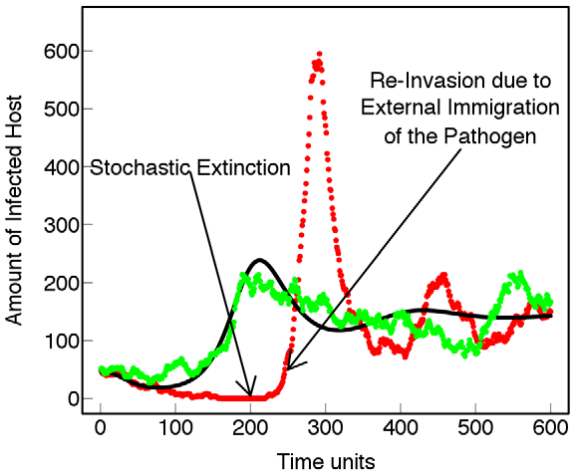
Stochasticity and extinction. Deterministic (black lines) and two stochastic realizations showing the temporal variation of the amount of infected host. In one case the uctuations lead to extinction while external immigration leads to re-invasion. *β* = 0.7*E*−4 *t* ^−1^.

**Figure 5:**
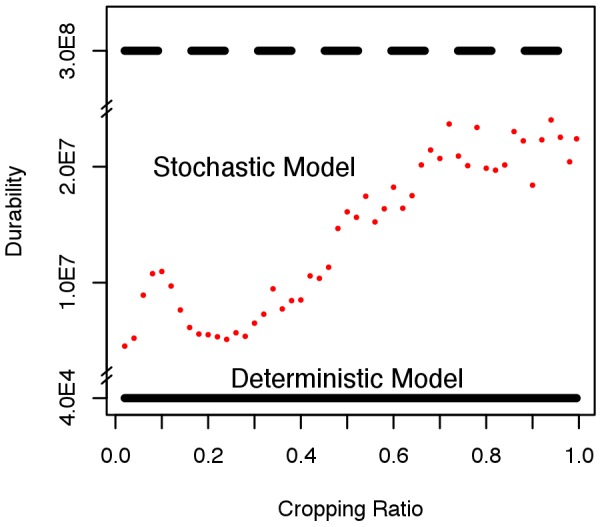
Stochasticity and durability of resistance. Durability of crop resistance *T_Add_ vs* cropping ratio. Stochastic case (red dots); deterministic case (black line). The black dashed line represents the largest theoretical 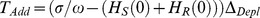 in the absence of the pathogen. Otherwise 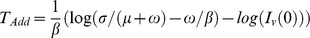 which coincides with the deterministic case. Data are averaged over 300 stochastic realizations. In the deterministic case the durability is practically unaffected by the cropping ratio. Larger proportion of the resistant cultivar enhances more frequent stochastic extinctions of the pathogen population therefore the durability increases with the cropping ratios (*β* = 7*E*−5, *σ* = 150, *μ* = 0.5).

Figure 3 shows that epidemics with the same basic reproductive number, 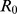, and the same 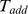, but with different pathogen life-cycle parameters, exhibit markedly different dynamics. The differences are expressed in the amplitudes of fluctuations, correlations and excursion times *i.e.* the time between an up-crossing (down-crossing) and subsequent down-crossing (up-crossing) relative to the deterministic profile. (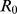 is defined as the expected number of secondary cases produced by a typical primary case in an entirely susceptible population, and depends solely on the life-cycle parameters). The importance of the intensity of such fluctuations is shown in Figure 4. Fluctuations might lead to a critically small infected population and subsequent stochastic extinction (and thus higher durability) while external immigration leads to re-invasion. In contrast with the predictions of [23] for a deterministic model, average durability of crop resistance 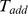 in the stochastic model increases with cropping ratio (Figure 5).

**Figure 6:**
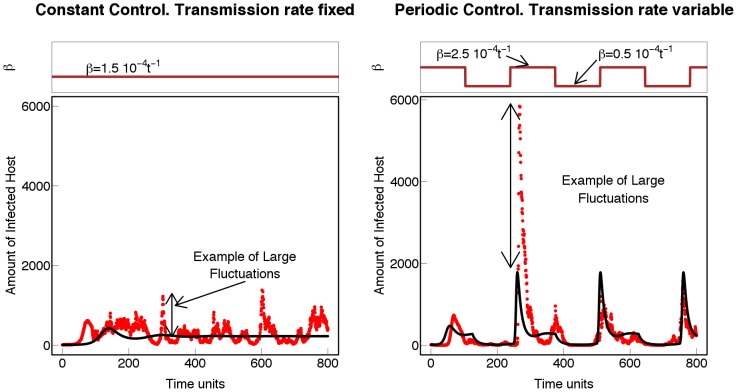
Resonance and epidemics. Deterministic (black line) and stochastic (red dots) profiles for the amount of infected host, no immigration. A) The transmission rate *β* is constant,i.e. no variable disease control is applied (brown line at the top of the figure). B) Resonance mechanism. Here we impose a wave-form transmission rate *β* with the same mean as in Figure 6.A (brown line at the top of the figure). This mimics the case when a periodic control is applied. When the frequency of the variable control is close to the natural frequencies of the pathosystem, the amplitudes of the stochastic uctuations dramatically increase.

**Figure 7:**
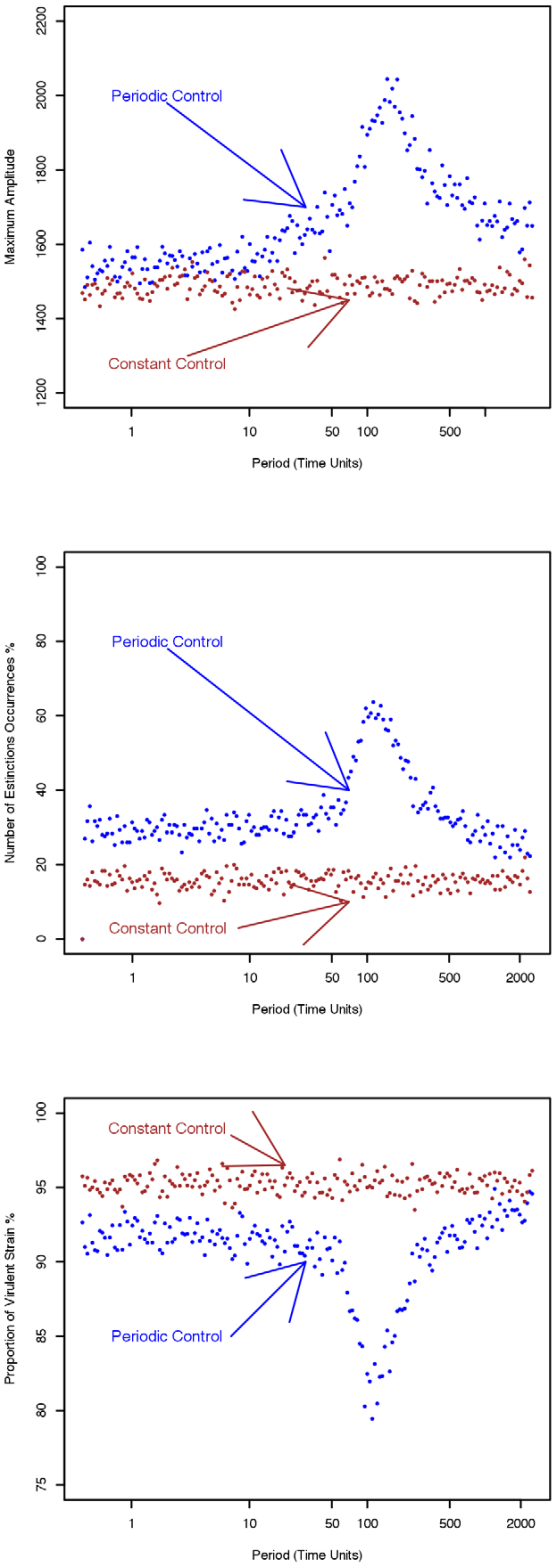
Effect of resonance on key epidemiological and evolutionary variables. **7**.A Amplitudes of the uctuations for the infected, virulent population. Blue circles: the maximum values of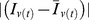, (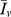 is the deterministic solution of equation (1)) occurring in the 300 simulated time-series *vs* the period of application of disease control, *i.e.* the period in the wave-form transmission rate *β* shown in Figure 6.B, compared with the case when constant control is applied, brown circles. 7.B Number of extinctions occurring in the simulated time-series *I_v_ vs* the period of application of disease control, blue circles, compared with the case when constant control is applied, brown circles. Since external immigration is allowed, more than one extinction can occur in the same time-series. Values are divided by the number of simulated time-series and expressed in percentage. 7.C Proportion of average, infected, virulent population. The number of 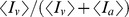 where 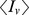 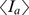
represent the time and ensemble average of the virulent and avirulent population *vs* the period of application of disease control, blue circles, compared with the case when constant control is applied, brown circles.

**Figure 8:**
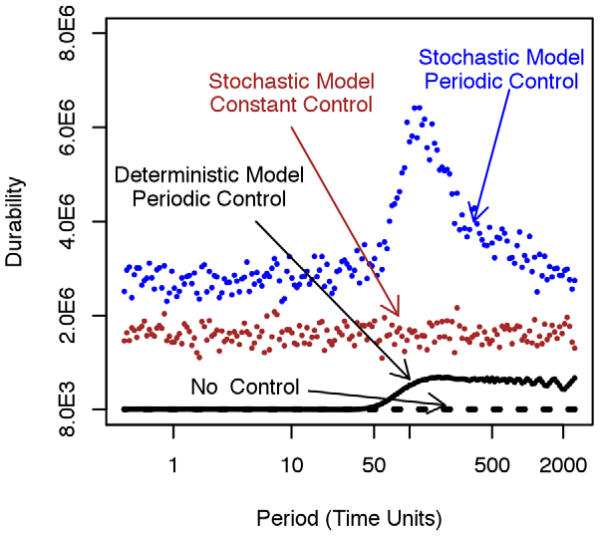
Resonance and durability of crop resistance. Durability of crop resistance *vs* the period of application of disease control compared with the case when constant control and no control is applied. Predictions for both stochastic and deterministic model are shown. This leads to three messages: 1) The durability predicted by the stochastic models is always larger than that predicted by the deterministic models 2) The durability is always larger when periodic control is applied 3) Stochasticity and periodicity lead to resonance with the natural frequencies of the pathosystem (maximum in the blue-dots profile) with strong effect on durability. The dashed black line represents predictions for the deterministic case in absence of control (≈8000), this is 1∶5 lower than the deterministic case in the presence of constant control but it is not shown the current plot as the two profiles are indistinguishable.

The existence of dominant frequencies from wavelet analysis are illustrated for the time-series of the population infected by the virulent strain (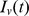) in the online Supporting Information. The dominant periods for stochastic realizations occur at ⪆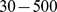 time units with a peak in the average wavelet spectrum in the range 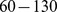 time units. The interaction of such dominant periods with a periodic perturbation, such as the intermittent application of fungicide control that affects the transmission rates (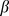), leads to large fluctuations in the infected population (cf Figure 6). This occurs in both the deterministic (black line) and stochastic (red dots) scenarios illustrated in Figure 6. However in the stochastic case the amplitudes of the fluctuations are, in general, larger than the corresponding deterministic case. The increased amplitudes are attributable to resonance with the natural frequencies of the system. We analyse the effect further by considering how two key epidemiological variables (maximum amplitude and number of extinctions) respond to changes in the periodic perturbation in applying chemical control (Figures 7.A and 7.B). In each case, the response effected by resonance with the periodic control dramatically increases in the region of dominant natural frequencies *i.e.* in the range 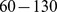 time units. Our analyses also show that the effects of resonance with periodic control can reduce the amount and the proportion of the virulent form in the population. Figure 7.C shows how the proportion of 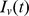 (averaged over realisations and time) changes with the period of control. The major effect corresponds once again with the dominant natural frequencies. The principal effects are summarised in Figure 8. Here we show that periodic (rather than constant) application of control increases the durability (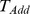) for both the deterministic and stochastic models. The effect is substantially enhanced however for the stochastic model with a marked response corresponding to the natural frequencies.

## Discussion

Using the stochastic formulation of the deterministic model proposed by van den Bosch and Gilligan [23] with allowance for immigration of avirulent and virulent strains, we have identified important differences in the behaviour of deterministic and stochastic models. Our results highlight the potential importance of stochastic fluctuations on the evolution of the pathogen and ultimately on the durability of crop resistance. The primary message is that large fluctuations can lead to a critically small infected population and subsequent stochastic extinction (see [31] for examples in the ecology literature). Stochastic extinction as described here is likely to be particularly important at the start of an epidemic, for a system with a large supply of healthy host and/or for diseases with small 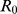. The results support the view that in the presence of random migration of pathogens from external fields, there is an intermittent scenario in which the pathosystem temporarily flips between three different regimes (“no invasion”, “invasion and no persistence”, “invasion and persistence” [32]). This has profound consequences for the durability of crop resistance since the overall measure of durability of crop resistance is determined by the total contribution of stochastic realisations comprising these three different regimes. The results support the two hypotheses posed in the introduction that in contrast to the finding of van den Bosch and Gilligan [23], the durability of crop resistance depends on the cropping ratio when we take account of demographic stochasticity. And a superimposed external source of stochasticity interacts with the intrinsic demographic fluctuations and affects the durability of resistance. We discuss each of these below.

### Existence of an optimal cropping ratio

The introduction of a resistant cultivar promotes more frequent stochastic extinctions of the pathogen population resulting in a higher 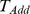 for larger cropping ratios (Figure 5). The reason is attributable to the occurrence of different equilibria depending upon whether the pathogen is present or absent. For simplicity we consider the case with no external immigration and no mutations, but the result still holds in the more general case. Ignoring random effects, in the absence of the pathogen, the densities of the resistant and susceptible cultivars approach the steady state given by 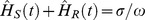, reflecting the planting and harvesting rates. When the pathogen is present, the system reaches an equilibrium in which the virulent genotype coexists with the resistant and susceptible cultivars. The equilibrium is given by 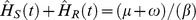 subject to 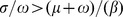 (see [23]). Thus depending upon whether the pathogen is present or not the behaviour of the pathosystem switches between these two states characterized by different equilibria. The larger the proportion of resistant crop, the smaller the density of infected hosts, especially at the beginning of an epidemic. When the density becomes critically low, the probability of extinction of the pathogen due to random fluctuations increases leading the system towards the steady state with larger yield, *i.e.* 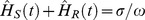. Therefore the stochastic profile for the durability of resistance (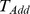) increases with the cropping ratio (Figure 5). The effect becomes more important when the gap between the two different equilibria is larger (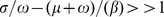).

Conversely, the effect is negligible when the average density of infected hosts is larger than the typical size of the fluctuations. This situation typically occurs for large initial proportions of the virulent strain or when the resistance is broken. The local minimum in the stochastic profile for 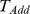 at low cropping ratio (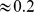) is an effect of the initial conditions. The latter were chosen as the equilibrium state in the absence of the resistant cultivar and no external immigration of the pathogen. At the beginning of the simulated epidemics, the external immigration of the avirulent pathogen causes an abrupt increase in the basic reproductive number. This, in turn, leads to a sharp decrease in the durability of resistance. The contribution becomes less important for large cropping ratio, since the resistant cultivar is immune to the avirulent strain. Allowance for immigration of the virulent strain has a negligible effect since it is several orders of magnitude smaller than the immigration of the avirulent strain. This response, suggesting the existence of an optimal cropping ratio (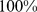 resistant), is in contrast to the predictions of van den Bosch and Gilligan [23]. However, the presence of disease resistance genes might lead to yield penalties [33]. By including a correction for yield penalty, we expect that the yield/durability would exhibit a maximum at an intermediate value of the cropping ratio.

### Resonance between environmental fluctuations and the intrinsic periodicity of the pathosystem

Agricultural systems are often subjected to periodic perturbation. Examples of such perturbations may arise from environmental forcing, for example temperature-driven changes in life-cycle parameters, which is particularly important as climate change has also been associated with variation in human and plant diseases [34], [35]. Another important source of periodic perturbations are temporal variations in disease control practices *e.g.* due to periodic changes in the mean dosage of applied fungicides, alternation of the application of protectant and curative fungicides. To this end Gubbins and Gilligan [36] developed deterministic and stochastic models to study fungicide resistance under the application of constant and periodically varying fungicides. They found the existence of a threshold for the invasion of the resistant strain that depended upon the relative fitness of the resistant strain and the effectiveness of control, which is turn is influenced by the periodicity of fungicide application [36]. Although it recognizes the importance of periodicity, the paper by [36] does not investigate how the periodicity of application interacts with the typical frequencies of the pathosystem *i.e.* resonance. Resonance is well documented in many biological and ecological systems ([31], [37]–[40] and references therein). Epidemics are characterized by particular frequencies rather than others: for example Grenfell *et al.* [41] (see also [42]) investigated synchrony patterns of measles in the UK and found that the epidemic time-series are dominated by a 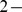
year periodic mode. Temporal patterns of epidemics, *e.g.* measles, whooping cough and cholera, are also linked with environmental and human changes (see [42] and references therein). In particular Rodo *et al.* [34] showed evidence of a relationship between El Niño/Southern Oscillation (ENSO) and cholera prevalence in Bangladesh. This suggests that such special frequencies might be present in plant disease epidemics too, although, to the authors' knowledge, long-term, high resolution time-series suitable for testing this hypothesis are not yet available in the literature. The existence of dominant frequencies for any epidemic described by an SIR-type mechanism is predicted by the theoretical analyses of closed systems by Alonso *et al.* [38] and Rozhnova and Nunes [43]. An important result for those models is that the frequency at which the power spectrum shows a peak, depends solely on the parameters underlying the disease dynamics. From a biological point of view, this suggests that the important time-scales arising from the fluctuations are expected to be related to the typical time-scales of the pathosystem (*e.g.* infectious period, lifetime of the infected individual). It also appears that the dominant periods increase with the population of the host, as both the amplitude and the periods of fluctuations become smaller at low population densities. An external, periodic, even small perturbation with the same frequency as the dominant frequency will resonate with the system resulting in large oscillations (Figure 7). In general this leads to extinction of the pathogen and hence to longer durability of crop resistance.

### Further challenges

We make a number of simplifying assumptions in our analyses in order to test the key hypotheses discussed above. Our conclusions are derived for a system with continuous availability of healthy hosts. We do this to avoid introducing additional periodicity into the system in which we are seeking to examine resonance between a particular external perturbation (periodic application of chemical control) and the intrinsic periodicity of the pathosystem. It is reasonable to suppose that seasonal availability in the supply of the host would affect the distribution of the dominant frequencies. It is also possible that the combination of large fluctuations arising from resonance and an upper limit of available host imposed by a carrying capacity for the crop could lead to large outbreaks of disease rather than to extinction. Detailed analyses of these effects are beyond the scope of the current paper but will be addressed in future work.

We have shown that the effect of stochasticity is important when the infected population is still low, which occurs at the beginning of the epidemics. This condition is always satisfied in a seasonal system at the beginning of each season, thus we can infer that including stochasticity in epidemic models is particularly important in seasonally variable crop management. In addition, the periodic forcing due to seasonality might interact with the dominant frequency in the absence of seasonality leading to intriguing dynamics (see *e.g.* [41], [44], [45]) which still need to be explored.

We have also neglected explicitly-spatial effects, except for external immigration. Spatial structure and synchrony are likely to affect patterns of population fluctuation. In particular, the spatial arrangements of resistant and susceptible fields in the landscape are likely to affect extinction and re-invasion, and hence durability. For example, Park *et al.* [46] have previously shown that extinction times for a single pathogen strain show a non-monotonic response as the size of the sub-population increases. The effect was shown to depend upon the transit time from arrival to leaving a patch. It is conceivable that the natural frequencies of the system are affected by the spatial arrangements and sizes of resistant and susceptible fields in the landscape and by the type of dispersal of the pathogen (long and short range rather than uniformly mixed as assumed here). Future research will seek to understand how the frequencies of the system depend upon the metapopulation parameters in order to establish whether or not there is an optimal patch size for the deployment of resistant cultivars.

Previous theoretical [19] and experimental [47], [48] work has investigated how individual components of the pathogen life-cycle affect the expression of crop resistance. This is particularly important for quantitative resistance, in which differing combinations of the components such as the infection efficiency, latent period, sporulation rate and infectious period, affect the expression of partial resistance [13], [18], [49]. We have shown in Figure 3 that different combinations of the parameters, representing different life-cycle components, can result in pathosystems characterized by the same 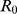 but different amplitudes of fluctuations. Despite the pioneering work of Alonso *et al.* [38] and Rozhnova and Nunes, [43], a complete understanding of the relationship between the dominant frequencies, life-cycle parameters, and other features of a pathosystem, such as spatial and temporal heterogeneities in parameter values, is still lacking. Further work is needed to tease out the effects of life cycle components on amplitudes of fluctuations and the importance of the results for plant breeders and the agrochemical industry to assist decisions over which specific pathogen life history traits to target in order to maximize the durability of crop or fungicide resistance.

Experimentation will of course be necessary to confirm or reject the hypotheses posed in this paper. An empirical test would require first i) to collect a long and high-resolution time-series of epidemiological data, perhaps starting with one cultivar only ii) to detect at which range the dominant frequency occurs [30], [42], [45] iii) then to apply a series of periodic perturbations with different periods. Even if direct measurements of durability is difficult, these kind of experiments ought to be able to detect variations in the amplitude of fluctuations (as in Figure 7.A), frequency of extinctions (as in Figure 7.B) for the different periods of perturbation. It is possible that such experiments could first be undertaken for experimental microcosms (cf [50]) to demonstrate a proof of concept.

## Supporting Information

Figure S1
**Wavelet analysis for the epidemic of infected, virulent population. Stochastic case.** A. Left, wavelet power spectrum of the root transformed time-series. Low values of the power spectrum are shown in dark blue, and high values in dark red. The dotted white lines show the maxima of the undulations of the wavelet power spectrum and the dotted-dashed black lines show the 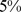 significant levels computed based on 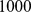 bootstrapped series. The light blue shaded areas identify the region influenced by edge. Right Average wavelet power spectrum. Panels B,C and D. As in A but for different stochastic realisations.(TIFF)

Figure S2 **Wavelet analysis for the epidemic of infected, virulent population. Deterministic case.** Colour scheme as in Figure S1. In the deterministic case the only relevant fluctuations occur at the beginning of the epidemic.
(TIFF)

Text S1
**Mathematical derivation of the stochastic model and wavelet analysis of the time-series** 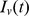 **.**
(PDF)
