# Supplementary figures and images for: Durable Resistance to Crop Pathogens: An Epidemiological Framework to Predict Risk under Uncertainty
Source: PLoS Comput Biol. 2013 Jan 17;9(1):e1002870. doi: 10.1371/journal.pcbi.1002870 (PMC3547817; doi:10.1371/journal.pcbi.1002870)

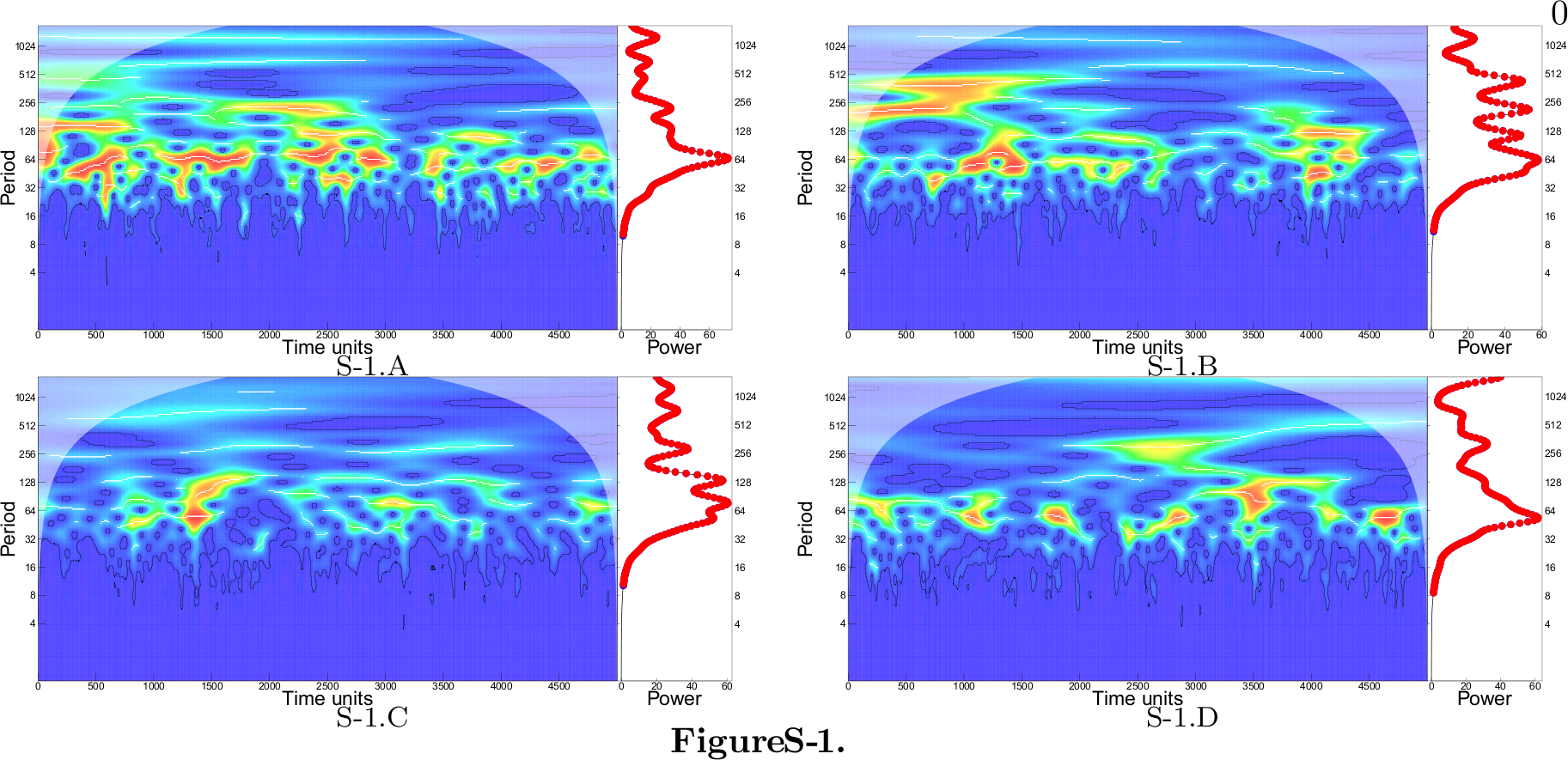

Supplement: Figure S1 — Wavelet analysis for the epidemic of infected, virulent population. Stochastic case. A. Left, wavelet power spectrum of the root transformed time-series. Low values of the power spectrum are shown in dark blue, and high values in dark red. The dotted white lines show the maxima of the undulations of the wavelet power spectrum and the dotted-dashed black lines show the significant levels computed based on bootstrapped series. The light blue shaded areas identify the region influenced by edge. Right Average wavelet power spectrum. Panels B,C and D. As in A but for different stochastic realisations. (TIFF) [file pcbi.1002870.s001.tiff]

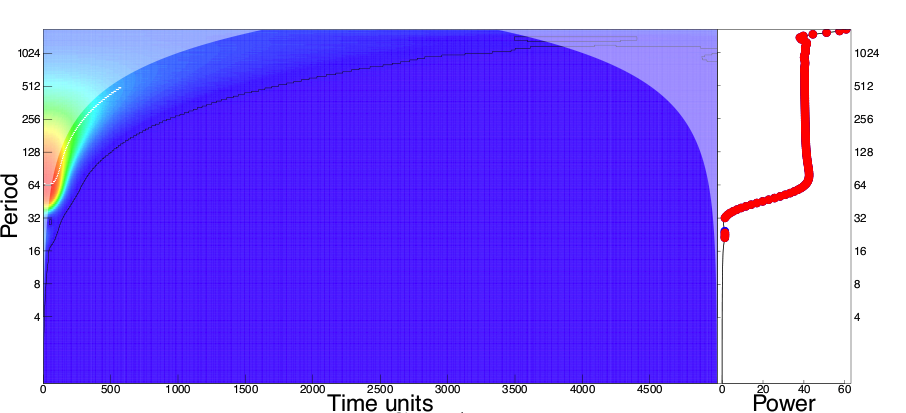

Supplement: Figure S2 — Wavelet analysis for the epidemic of infected, virulent population. Deterministic case. Colour scheme as in Figure S1. In the deterministic case the only relevant fluctuations occur at the beginning of the epidemic. (TIFF) [file pcbi.1002870.s002.tiff]
