# Supplementary material for: Durable Resistance to Crop Pathogens: An Epidemiological Framework to Predict Risk under Uncertainty
Source: PLoS Comput Biol. 2013 Jan 17;9(1):e1002870. doi: 10.1371/journal.pcbi.1002870 (PMC3547817; doi:10.1371/journal.pcbi.1002870)
Supplement: Text S1 — Mathematical derivation of the stochastic model and wavelet analysis of the time-series . (PDF) [file pcbi.1002870.s003.pdf]

Supporting Information. Text S1

Mathematical details for the Stochastic model

The infection is transmitted by spores generated from an individual and being in contact with another individual and it is described by the following process (where we ignore the planting and harvest process):

$$\begin{aligned}
&I_a + H_S \xrightarrow{(1-\epsilon)\beta} 2I_a \\
&I_v + H_S \xrightarrow{(1-\epsilon)\beta} 2I_v \\
&I_v + H_R \xrightarrow{(1-\epsilon)\beta} 2I_v \\
&I_a + H_S \xrightarrow{\epsilon\beta} I_a + I_v \\
&I_a + H_R \xrightarrow{\epsilon\beta} I_a + I_v \\
&I_v + H_S \xrightarrow{\epsilon\beta} I_a + I_a \\
&I_a \xrightarrow{\mu} R_a \\
&I_v \xrightarrow{\mu} R_v
\end{aligned} \tag{S-1}$$

where the formalism $n_1P_1 + n_2P_2 \xrightarrow{\pi} m_1Q_1 + m_2Q_2$ means that n_1 individuals from the population P_1 encountering n_2 individuals from the population P_2 will generate m_1 individuals of population Q_1 and m_2 individuals of population Q_2 with probability of transition π. From the transition rates in (S-1) , one can write the Master equation; as the total population $I_a + I_v + H_S + H_R$ is large, then we can apply the Kramer-Moyal expansion leading to the expression for the matrix A (drift term) and B (diffusion term) in the resulting Fokker-Planck equation [1, 2] .

$$A = \begin{pmatrix} -t_1^+ - t_2^+ - t_4^+ - t_6^+ \\ -t_3^+ - t_5^+ \\ t_1^+ + t_6^+ - t_7^+ \\ t_2^+ + t_3^+ + t_4^+ + t_5^+ - t_8^+ \\ t_7^+ \\ t_8^+ \end{pmatrix}$$

$$\mathbf{B} = \begin{pmatrix} t_1^+ + t_2^+ + t_4^+ + t_6^+ & 0 & -t_1^+ - t_6^+ & -t_4^+ - t_2^+ & 0 & 0 \\ 0 & t_3^+ + t_5^+ & 0 & -t_3^+ - t_5^+ & 0 & 0 \\ -t_1^+ - t_6^+ & 0 & t_1^+ + t_6^+ + t_7^+ & 0 & -t_7^+ & 0 \\ -t_2^+ - t_4^+ & -t_3^+ - t_5^+ & 0 & t_2^+ + t_3^+ + t_4^+ + t_5^+ + t_8^+ & 0 & -t_8^+ \\ 0 & 0 & -t_7^+ & 0 & t_7^+ & 0 \\ 0 & 0 & 0 & -t_8^+ & 0 & t_8^+ \end{pmatrix} \tag{S-2}$$

where

$$\begin{aligned} t_i^- &= 0 \quad i = 1, 8 \\ t_1^+ &= (1-\epsilon)\beta H_S I_a \\ t_2^+ &= (1-\epsilon)\beta H_S I_v \\ t_3^+ &= (1-\epsilon)\beta H_R I_v \\ t_4^+ &= \epsilon\beta H_S I_a \\ t_5^+ &= \epsilon\beta H_R I_a \\ t_6^+ &= \epsilon\beta H_S I_v \\ t_7^+ &= \mu I_a \\ t_8^+ &= \mu I_v \end{aligned} \tag{S-3}$$

The deterministic terms in the corresponding Langevin equation are given by the matrix A, while the stochastic terms are obtained by the decomposition of the diffusion matrix $\mathbf{C}\mathbf{C}^T = \mathbf{B}$ by using the Cholesky algorithm. The multidimensional Langevin equation for the stochastic model is:

$$\begin{aligned} \mathrm{d}H_S &= (1-\phi)\sigma \mathrm{d}t - \omega H_S(t)\mathrm{d}t - \beta H_S(I_a + I_v)\mathrm{d}t + \sum_{j=1}^{6} c_{1,j}\mathrm{d}W_j(t) \\ \mathrm{d}H_R &= \phi\sigma \mathrm{d}t - \omega H_R(t)\mathrm{d}t - H_R[(1-\epsilon)I_v + \epsilon I_a]\mathrm{d}t + \sum_{j=1}^{6} c_{2,j}\mathrm{d}W_i(t) \\ \mathrm{d}I_a &= -(\mu+\omega)I_a \mathrm{d}t + \beta H_S[(1-\epsilon)I_a + \epsilon I_v]\mathrm{d}t + \sum_{j=1}^{6} c_{3,j}\mathrm{d}W_j(t) \\ \mathrm{d}I_v &= -(\mu+\omega)I_v \mathrm{d}t + \beta\{H_S[(1-\epsilon)I_v + \epsilon I_a] + H_R[(1-\epsilon)I_v + \epsilon I_a]\}\mathrm{d}t + \sum_{j=1}^{6} c_{4,j}\mathrm{d}W_j(t) \\ \mathrm{d}R_a &= (\mu-\omega)R_a \mathrm{d}t + \sum_{j=1}^{6} c_{5,j}\mathrm{d}W_j(t) \\ \mathrm{d}R_v &= (\mu-\omega)R_v \mathrm{d}t + \sum_{j=1}^{6} c_{6,j}\mathrm{d}W_j(t) \end{aligned} \tag{S-4}$$

where $W_i(t)$ is a Wiener process with mean zero and variance dt and $c_{i,j}$ are the elements of the matrix C.

As a consequence of Kramer-Moyal expansion, the fluctuations around the mean values are described by a Gaussian distribution. This is not strictly true when one or more categories (*e.g.* the infected population) are close to extinction since the fluctuations must be governed by a skewed distribution. However, the intensities of the fluctuations are expected to decrease with the mean values (*i.e.* smaller variance of the Gaussian distribution) and therefore the inaccuracy of the approximation is expected to be negligible, at least for our purposes. The accuracy of the model can be increased by employing higher order terms in the Kramer-Moyal expansion.

Wavelet analysis of the time-series $I_v(t)$.

Here we show the wavelet power spectrum [3–5] for four single randomly chosen, realizations of the non-stationary time-series $I_v(t)$, representing the population infected by the virulent strain of the pathogen (Figure S-1). All random realizations show similar patterns. Prediction for the deterministic case is also shown for comparison (Figure S-2).

A key message is that in general resonance leads to large fluctuations, these lead to extinction if the population of infected is small, which typically occur at the beginning of the epidemics. Thus the probability of extinction is expected to be affected more strongly by the initial behaviour of the power spectrum (right side of Figure S-2) compared with the portion of spectrum at long times.

References

1. Gardiner CW (2004) Handbook of stochastic methods for physics, chemistry, and the natural sciences. Berlin ; London: Springer, 3rd ed. edition.

2. Kampen NGv (2007) Stochastic processes in physics and chemistry. North-Holland personal library. Amsterdam ; London: Elsevier, 3rd ed. edition.

3. Cazelles B, Chavez M, Berteaux D, Ménard F, Vik JO, et al. (2008) Wavelet analysis of ecological time series. Oecologia 156: 287–304.

4. Cazelles B, Chavez M, de Magny GC, Guegan JF, Hales S (2007) Time-dependent spectral analysis of epidemiological time-series with wavelets. Journal of the Royal Society Interface 4: 625–636.

5. Aldrich E (2012) A package of functions for computing wavelet filters, wavelet transforms and multiresolution analyses. (R package)
`http://cran.r-project.org/web/packages/wavelets/index.html`

Figure S-1: **Wavelet analysis for the epidemic of infected, virulent population. Four different stochastic realisation.** For each subfigure: Left, wavelet power spectrum of the root transformed time-series. Low values of the power spectrum are shown in dark blue, and high values in dark red. The dotted white lines show the maxima of the undulations of the wavelet power spectrum and the dotted-dashed black lines show the 5% significant levels computed based on 1000 bootstrapped series. The light blue shaded areas identify the region influenced by edge. Right Average wavelet power spectrum.

Figure S-2: **Wavelet analysis for the epidemic of infected, virulent population. Deterministic case** Colour scheme as in Figure S-1. In the deterministic case the only relevant fluctuations occur at the beginning of the epidemic.
